# Optimizing the timing of an end-of-outbreak declaration: Ebola virus disease in the Democratic Republic of the Congo

**DOI:** 10.1126/sciadv.ado7576

**Published:** 2024-07-03

**Authors:** William S. Hart, Jack M. Buckingham, Mory Keita, Steve Ahuka-Mundeke, Philip K. Maini, Jonathan A. Polonsky, Robin N. Thompson

**Affiliations:** ^1^ Wolfson Centre for Mathematical Biology, Mathematical Institute, University of Oxford, Oxford OX2 6GG, UK.; ^2^ EPSRC Centre for Doctoral Training in Mathematics for Real-World Systems, Mathematics Institute, University of Warwick, Coventry CV4 7AL, UK.; ^3^ World Health Organization, Regional Office for Africa, Brazzaville, Republic of the Congo.; ^4^ Institute of Global Health, Faculty of Medicine, University of Geneva, Geneva 1202, Switzerland.; ^5^ Institut National de Recherche Biomédicale, Kinshasa, Democratic Republic of the Congo.; ^6^ Geneva Centre of Humanitarian Studies, University of Geneva, Geneva 1205, Switzerland.

## Abstract

Following the apparent final case in an Ebola virus disease (EVD) outbreak, the decision to declare the outbreak over must balance societal benefits of relaxing interventions against the risk of resurgence. Estimates of the end-of-outbreak probability (the probability that no future cases will occur) provide quantitative evidence that can inform the timing of an end-of-outbreak declaration. An existing modeling approach for estimating the end-of-outbreak probability requires comprehensive contact tracing data describing who infected whom to be available, but such data are often unavailable or incomplete during outbreaks. Here, we develop a Markov chain Monte Carlo–based approach that extends the previous method and does not require contact tracing data. Considering data from two EVD outbreaks in the Democratic Republic of the Congo, we find that data describing who infected whom are not required to resolve uncertainty about when to declare an outbreak over.

## Teaser

Contact tracing data are not required to determine when to declare Ebola outbreaks over.

## INTRODUCTION

Ebola virus infections have severe consequences, with case fatality rates between 25 and 90% in different Ebola virus disease (EVD) outbreaks (*1*). The World Health Organization (WHO) therefore recommends applying combinations of public health and social measures (PHSM) to bring outbreaks under control quickly, including surveillance, case isolation, contact tracing, safe and dignified burials, vaccination, and community engagement and risk communication (*1*).

Following the final recorded case in an outbreak of EVD (or another severe disease that necessitates stringent interventions), a key consideration is when the outbreak can be declared over safely and PHSM relaxed without a substantial risk of additional cases occurring. WHO guidance for EVD recommends that the acute phase of the outbreak can be declared over if no further cases are detected for a period of 42 days (twice the theoretical maximum incubation period for Ebola virus infection) following the last potential exposure to a recorded case (*2*). However, mathematical modeling studies have indicated that the probability of no future cases occurring (the end-of-outbreak probability) depends not only on the time since the last case but also on features of the specific outbreak under consideration (*3*–*5*). Estimates of the end-of-outbreak probability have therefore been proposed as an alternative basis for determining the timing of an end-of-outbreak declaration (*3*–*5*).

In previous work (*5*), we developed an approach for calculating the end-of-outbreak probability that can be used when the outbreak’s transmission tree (i.e., data describing who infected whom) is available. The approach in (*5*) allows for exact calculation of the end-of-outbreak probability using the branching process model underlying a widely used (*6*–*11*) approximate method developed by Nishiura *et al.* (*6*). However, the transmission tree typically requires substantial contact tracing resources to construct and is rarely available in complete form during outbreaks.

Here, building upon the approach introduced in (*5*), we develop a method for inferring the end-of-outbreak probability exactly under the same branching process model, but without requiring the transmission tree to be known. Specifically, we show how the method from (*5*) can be combined with data augmentation Markov chain Monte Carlo (MCMC) (*12*–*15*) to estimate and account for uncertainty in the transmission tree.

We apply our MCMC-based approach to obtain quasi–real-time estimates of the end-of-outbreak probability using data from two historical EVD outbreaks in the Democratic Republic of the Congo (DRC). First, we show that this method gives similar results to the approach from (*5*), but without requiring knowledge of the outbreak transmission tree, by applying both methods to data from a small outbreak in the Likati health zone in 2017 for which the transmission tree is available (*16*, *17*). Then, we go on to apply the MCMC approach to disease incidence data from a larger outbreak in Équateur province in 2020 (*18*, *19*). We find that knowledge of the transmission tree of this outbreak would have been unlikely to affect model-based determination of the timing of an end-of-outbreak declaration substantially. In addition, our analyses suggest that both outbreaks could theoretically have been declared over earlier than the actual dates on which end-of-outbreak declarations were made (based on the WHO’s 42-day guideline) with only a small risk of further cases occurring.

## RESULTS

**Fig. 1.**
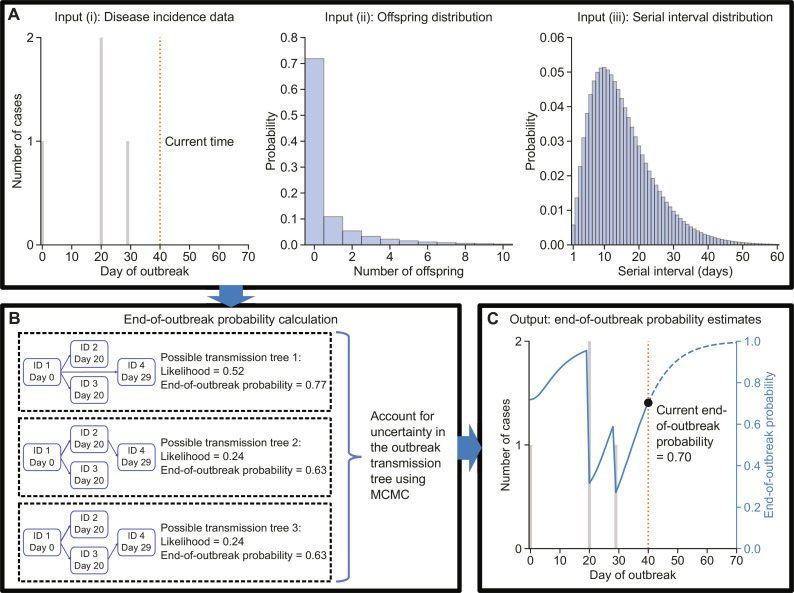
Schematic illustrating our MCMC-based approach for estimating the end-of-outbreak probability. (**A**) The MCMC method for calculating the end-of-outbreak probability requires three inputs: disease incidence time series data [input (i)], the offspring distribution [input (ii)], and the serial interval distribution [input (iii)]. The offspring and serial interval distributions shown are the actual distributions for EVD used in our analyses (see Materials and Methods). (**B**) The likelihoods of different possible transmission trees being the true transmission tree given the disease incidence data (up to the current time) are derived here, and the end-of-outbreak probability conditioned on a particular transmission tree can be calculated using the traced method introduced in (*5*). The end-of-outbreak probability given inputs (i) to (iii) can be calculated either by combining the end-of-outbreak probabilities under every possible transmission tree, weighted by the likelihood of each tree (enumerate method), or, equivalently, by using MCMC to sample possible transmission trees from the likelihood (MCMC method). (**C**) End-of-outbreak probability estimates on each day during and following the outbreak can be obtained by applying this process using data up to and including the current day.

Our MCMC-based approach for estimating the end-of-outbreak probability (hereafter referred to as the MCMC method) is illustrated in Fig. 1. The MCMC method requires three inputs (Fig. 1A): (i) disease incidence time series data (i.e., daily or weekly counts of newly identified cases); (ii) the offspring distribution (the probability distribution characterizing the number of secondary cases generated by an infected host); and (iii) the serial interval distribution (the probability distribution describing the interval between the symptom onset times of an infector-infectee transmission pair). As shown in (*5*), if the outbreak’s transmission tree is also available (up to the current time), then the end-of-outbreak probability can be derived analytically under a branching process transmission model [the traced transmission method in (*5*), here shortened to the traced method]. If the transmission tree is unknown, then (as shown in Materials and Methods) the likelihoods of different transmission trees (that are consistent with the disease incidence time series data) being the true transmission tree can be derived under the same branching process transmission model (Fig. 1B). The end-of-outbreak probability given the disease incidence data is then given by a sum, taken over all possible transmission trees, of the end-of-outbreak-probability conditional on each tree (calculated using the traced method), weighted by the likelihood of that tree (we refer to the direct evaluation of this sum by enumerating all possible transmission trees as the enumerate method).

However, for all but very small outbreaks, evaluating this sum directly requires a large number of possible transmission trees to be considered. Therefore, in the MCMC method, we instead use MCMC to obtain a sample of possible transmission trees from the likelihood, which can then be used to calculate an estimate of the end-of-outbreak probability that is equivalent to that from the enumerate method (in the limit of a large number of MCMC iterations). Details of the different methods for estimating the end-of-outbreak probability are given in Materials and Methods.

### Simulation study

**Fig. 2.**
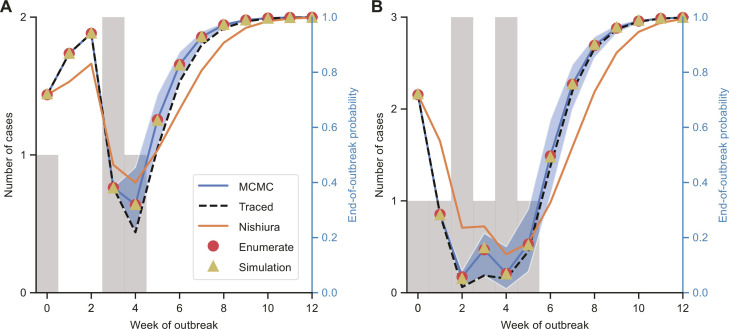
End-of-outbreak probability estimates for simulated datasets with weekly data. (**A**) Disease incidence data (gray bars; values are shown on the left *y* axis) and end-of-outbreak probability estimates obtained using the MCMC, traced, Nishiura, enumerate, and simulation methods (values are shown on the right *y* axis) for the first simulated dataset. The blue shaded region indicates 95% credible intervals of end-of-outbreak probability estimates obtained in individual MCMC iterations. (**B**) Equivalent results for the second simulated dataset.

Before considering real-world EVD outbreak data, we first conducted analyses using synthetic data. This allowed us to not only compare outputs from the traced and MCMC methods in an idealized setting in which the true outbreak transmission tree is known, but also test our approach using data from multiple simulated outbreaks. Initially, we generated two small simulated datasets with weekly disease incidence data (Fig. 2) using offspring and weekly serial interval distributions representative of EVD transmission (fig. S1). Using small datasets with weekly data enabled us to verify in a simple setting that the MCMC method gives almost identical end-of-outbreak probability estimates to the enumerate method and to another theoretically equivalent (but more computationally demanding) approach: the simulation method, which involves estimating the end-of-outbreak probability by running a large number of simulations of the transmission model and then calculating, out of all simulations that match the observed disease incidence up to the current time, the proportion of those simulations in which no further cases occur.

For one of the simulated datasets (Fig. 2A), the MCMC method gives slightly higher end-of-outbreak probability estimates following the final case than the traced method, whereas for the other dataset, estimates from the two methods following the final case are very similar (Fig. 2B). Nonetheless, uncertainty in end-of-outbreak probability estimates obtained using the MCMC method, depending on the exact outbreak transmission tree, can be quantified: the blue shaded regions in Fig. 2 show 95% credible intervals of end-of-outbreak probability estimates in individual MCMC iterations (i.e., credible intervals for the possible end-of-outbreak probability values that could be obtained if the transmission tree was constructed via contact tracing).

In addition, we calculated end-of-outbreak probability estimates using an existing approach (*6*–*11*) (the Nishiura method), which is based on the same branching process transmission model as the MCMC method and requires the same inputs, but only provides an approximation to the end-of-outbreak probability under this transmission model (whereas the MCMC method infers the exact end-of-outbreak probability under that model; see Materials and Methods). For both outbreaks, estimates using the Nishiura method after the final recorded case are lower than those using the MCMC and traced methods, and are also below the lower limit of each 95% credible interval obtained using the MCMC method.

We also considered three further, larger, simulated datasets with daily, rather than weekly, disease incidence data (fig. S2). Because these simulated datasets are much larger and more detailed than those shown in Fig. 2 (comprising 36, 61, and 514 EVD cases), the enumerate and simulation methods were computationally challenging to apply. We therefore applied the MCMC and traced methods, and found that, for each dataset, the MCMC method gives comparable end-of-outbreak probability estimates following the final case to the traced method (but without requiring the outbreak transmission tree to be known). Furthermore, both of those methods again provide different estimates to the approximate Nishiura method. Example MCMC trace plots for end-of-outbreak probability calculations using the MCMC method are shown in fig. S3 for the largest simulated dataset.

As a final analysis of synthetic data, we tested the performance of the MCMC method when applied in scenarios with different transmission patterns at the end of the outbreak. Specifically, we compared end-of-outbreak probability estimates between the MCMC and traced methods for datasets in which a final case, which is an imported case, occurs after a range of possible time intervals following the penultimate case (fig. S4). We again found that the two methods give similar end-of-outbreak probability estimates, even if the final case is erroneously assumed to have arisen due to local transmission from previous cases when the MCMC method is used (fig. S4, D to F).

### EVD outbreak case studies

**Fig. 3.**
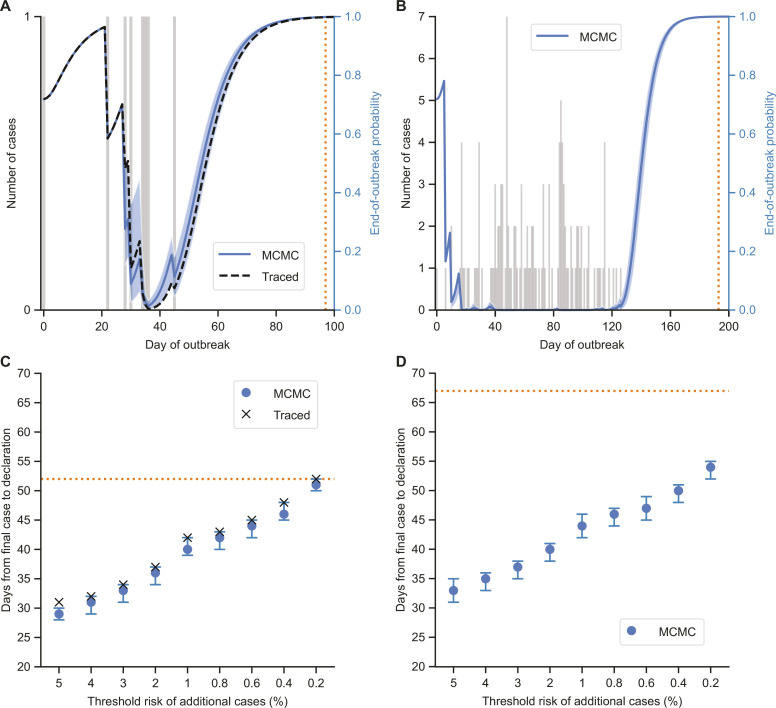
End-of-outbreak probability estimates for historical EVD outbreaks in the DRC. (**A**) Disease incidence data (gray bars; values are shown on the left *y* axis) and end-of-outbreak probability estimates obtained using the MCMC and traced methods (values are shown on the right *y* axis) for the 2017 EVD outbreak in Likati health zone, DRC. The blue shaded region indicates 95% credible intervals of end-of-outbreak probability estimates obtained in individual MCMC iterations. (**B**) Equivalent panel to (A) for the 2020 EVD outbreak in Équateur province, DRC (end-of-outbreak probabilities are only shown for the MCMC method, because the transmission tree was unavailable for this outbreak). (**C**) The earliest day following the day the final recorded case developed symptoms on which the 2017 Likati health zone outbreak could have been declared over, based on the percentage risk of further cases calculated using the MCMC or traced method falling below each of a range of threshold values. The error bars indicate 95% credible intervals of theoretical declaration dates obtained in individual MCMC iterations. (**D**) Equivalent panel to (C) for the 2020 Équateur province outbreak. In all panels, the actual day on which the outbreak was declared over (for both outbreaks, this was 42 days after the final case recovered) is indicated by an orange dotted line [vertical in (A) and (B) and horizontal in (C) and (D)]. Example MCMC trace plots for end-of-outbreak probability calculations using the MCMC method are shown in figs. S6 and S7.

We then applied the MCMC method to data from two historical EVD outbreaks in the DRC (Fig. 3). First, we considered an outbreak of eight EVD cases in the Likati health zone in 2017 (*16*, *17*) (Fig. 3A). Because the transmission tree for this outbreak is available (*17*) (fig. S5), we were able to compare end-of-outbreak probability estimates calculated using the MCMC and traced methods for this real-world outbreak (Fig. 3A). Then, we applied the MCMC method to disease incidence data from a larger outbreak of 130 EVD cases in Équateur province in 2020 (*18*–*20*) (Fig. 3B).

The MCMC method indicates that the risk of further cases occurring (i.e., one minus the estimated end-of-outbreak probability) on the dates each outbreak was actually declared over based on the 42-day guideline (these dates are given by the orange dotted vertical lines in Fig. 3, A and B) was low: 0.12% for the Likati health zone outbreak on 2 July 2017 (the corresponding risk value obtained using the traced method is 0.18%) and 0.01% for the Équateur province outbreak on 18 November 2020. This suggests that an earlier end-of-outbreak declaration could theoretically have been made for both outbreaks with only a small risk of a resurgence in cases. To explore this further, we considered when the two outbreaks could have been declared over, if a declaration was instead made based on the risk of additional cases occurring (obtained using the MCMC method or, for the Likati health zone outbreak only, the traced method) falling below a chosen threshold (Fig. 3, C and D). We also calculated 95% credible intervals of dates on which the outbreaks could have been declared over for the MCMC method, accounting for uncertainty in the outbreak transmission tree (error bars in Fig. 3, C and D).

The theoretical end-of-outbreak declaration date depends on both the risk threshold used and characteristics of the specific outbreak under consideration—for example, for a 1% risk threshold, the MCMC method suggests that the Likati health zone outbreak could have been declared over on 20 June 2017 (40 days after the final recorded case developed symptoms, and 12 days before the outbreak was actually declared over) and the Équateur province outbreak could have been declared over on 26 October 2020 (44 days after the final recorded case developed symptoms, and 23 days before the outbreak was actually declared over). For the Likati health zone outbreak, over all thresholds considered, the maximum difference in theoretical declaration dates between the MCMC method and the traced method is 2 days. For both outbreaks, the width of the 95% credible interval of the theoretical declaration date for the MCMC method (accounting for uncertainty in the transmission tree) is at most 4 days. These findings suggest that there may be limited benefit gained from collecting comprehensive contact tracing data solely to refine the timing of an end-of-outbreak declaration (Fig. 3D).

## DISCUSSION

Estimates of the probability that an EVD outbreak has ended can help to guide the timing of an end-of-outbreak declaration, enabling policy-makers to relax stringent and expensive PHSM as soon as possible without incurring a substantial risk of further cases. Here, we have built on previous work (*5*) to develop an MCMC-based modeling framework for estimating the end-of-outbreak probability. Unlike the traced method from (*5*), the MCMC method does not require information about who infected whom (i.e., the outbreak transmission tree) to be known. Applying our approach to two historical EVD outbreaks in the DRC, we found that both outbreaks could potentially have been declared over earlier than the actual end-of-outbreak declaration dates with only a small risk of further cases occurring.

As noted above, the major advantage of the MCMC method over the traced method is that the MCMC method does not require the outbreak transmission tree to be known. Intensive contact tracing typically conducted as part of EVD outbreak responses (*1*) may enable transmission trees to be constructed, particularly for smaller outbreaks, as was the case for the 2017 Likati health zone outbreak considered here (*17*). Because the traced method is straightforward to apply and leverages more data than the MCMC method, we continue to recommend that this method be used whenever the transmission tree is known to ensure that the most accurate possible end-of-outbreak probability estimates are obtained. However, the primary purpose of contact tracing is not to construct the complete outbreak transmission tree (but rather to identify and curtail chains of transmission), and particularly for larger outbreaks and/or in real time, the full transmission tree is not usually available. Our results demonstrate that the MCMC method can give similar results to the traced method while requiring only disease incidence time series data to be available (in addition to the offspring and serial interval distributions).

In addition to providing an overall end-of-outbreak probability estimate, the MCMC method can be used to quantify uncertainty in the end-of-outbreak probability depending on the (unknown) outbreak transmission tree. This uncertainty could potentially be factored into decisions about the timing of end-of-outbreak declarations, or used to evaluate the benefit of constructing the outbreak transmission tree through intensive contact tracing for refining end-of-outbreak probability estimates. For example, we found that knowledge of the transmission tree for the relatively large Équateur province outbreak would have been unlikely to alter the timing of an end-of-outbreak declaration based on end-of-outbreak probability estimates by more than 2 days (Fig. 3D).

We note that a range of alternative approaches exist for estimating the end-of-outbreak probability from disease incidence time series data (*4*, *6*–*11*, *21*–*26*). In particular, the most commonly used approach (*6*–*11*)—the Nishiura method—uses the same inputs as the MCMC method and is straightforward to apply. However, the Nishiura method only approximates the end-of-outbreak probability, even assuming that the underlying branching process transmission model is correct (see Materials and Methods), whereas the MCMC method infers the exact end-of-outbreak probability under the same transmission model. When we analyzed synthetic transmission data (Fig. 2 and fig. S2), we found substantial differences in end-of-outbreak probability estimates between the MCMC and Nishiura methods. This discrepancy is consistent with the findings in (*5*), and likely arises because the approximating assumption underlying the Nishiura method does not hold when extensive superspreading can occur [see (*5*) for details], as assumed for EVD here based on previous findings (*27*).

Our analyses of real-world and simulated data demonstrate that the MCMC method can be applied to EVD datasets of a range of sizes. In particular, the size of the largest simulated dataset that we considered (514 cases; fig. S2C) exceeds the number of reported cases in all but two historical EVD outbreaks (*28*). Application of the MCMC method to an entire disease incidence time series from a larger EVD outbreak with thousands of cases may require substantial computational resources. However, we note that large outbreaks are typically composed of multiple smaller localized outbreaks, and the MCMC method could be applied to these individual local outbreaks. A policy-maker could then choose to declare the large outbreak over when the risk of future cases arising from all local outbreaks is sufficiently low.

As in any modeling study, there are limitations to our results. First, our quantitative findings are only valid under the assumed transmission model and the input EVD offspring and serial interval distributions. However, estimates of these distributions differ between EVD outbreaks (*29*), potentially because of differences in Ebola virus subtype, interventions, and host behavior. Therefore, for practical use of our method, estimates specific to the outbreak under consideration should be used whenever possible. Because there may be substantial uncertainty in these distributions, particularly for small outbreaks, generalizing the MCMC method to account for this uncertainty (alongside uncertainty in the transmission tree) in end-of-outbreak probability estimates would be a useful future extension of our work. Temporal changes in the offspring and serial interval distributions could also be considered in a future study, as well as incorporation of partial transmission tree data (when available).

Another caveat of our study is that none of the methods for estimating the end-of-outbreak probability considered here account for under-ascertainment of cases or delays in reporting. Because these are likely to be important considerations for EVD, particularly when estimating the end-of-outbreak probability in real time to inform end-of-outbreak declarations (*2*, *3*, *8*, *22*, *30*), extending our MCMC method to account for these factors is a target for future research. Nonetheless, we emphasize that the MCMC method relaxes key limitations of existing approaches that also do not account for under-reporting and/or delayed reporting (the traced and Nishiura methods).

In summary, we have developed an MCMC-based approach for estimating the end-of-outbreak probability using disease incidence data. This method builds on a previous one (*5*), with the crucial difference being that the approach here does not require information about the outbreak transmission tree. Because the outbreak transmission tree is often unknown, this is a key extension to that previous research. The MCMC method suggests that two past EVD outbreaks could have been declared over earlier than the actual end-of-outbreak declaration dates determined using existing WHO guidelines (based on a period of 42 days passing without cases, following individuals last potentially being exposed to the previous case). In addition, if the outbreaks were instead declared over as soon as the estimated risk of further cases fell below a specified low threshold value, we found that lack of knowledge of the outbreak transmission tree would not have contributed substantial uncertainty to the theoretical end-of-outbreak declaration date. Consequently, comprehensive contact tracing may be unnecessary for the sole purpose of guiding the timing of an end-of-outbreak declaration. While EVD was our main focus here, the MCMC method can also be applied during outbreaks of other severe diseases. We hope that this will allow for informed public health decision-making about when stringent PHSM can be relaxed or removed.

## MATERIALS AND METHODS

### Transmission model

We considered a branching process transmission model characterized by two probability distributions:

1) The offspring distribution (the distribution of the number of secondary cases generated by each infected individual), with probability mass function *p*(*y*) for *y* = 0,1,2, ….

2) The discrete (daily or weekly) serial interval distribution (the distribution of the number of days or weeks between the symptom onset times of an infector-infectee pair), with probability mass function *f*(*x*) and cumulative distribution function *F*(*x*) for *x* = 1,2,3, …. We assumed that the serial interval can only take strictly positive values, which is a reasonable assumption for EVD (with daily data) as presymptomatic transmission is uncommon (*31*).

Specifically, in the forward transmission model, the number of secondary cases generated by each infected individual is sampled from the offspring distribution, and the symptom onset times of those secondary cases are then sampled according to the serial interval distribution (independently for each case).

The methods for calculating the end-of-outbreak probability described below could, in principle, be applied using any offspring distribution. However, as described below, some analytic simplifications are possible under the assumption of a negative binomial offspring distribution with mean *R* (the reproduction number) and dispersion parameter *k* [which characterizes the extent of superspreading, with a lower value of *k* corresponding to a greater degree of superspreading (*32*)]. In this case, the probability mass function of the offspring distribution is

$$p\left(y\right)=\frac{\Gamma \left(k+y\right)}{y!\Gamma \left(k\right)}q^{y}{\left(1-q\right)}^{k},\;\text{for}\;y=0,1,2,\ldots$$

where we define *q* = *R*/(*R* + *k*).

The offspring and serial interval distributions used in our analyses are described below (see the “EVD offspring and serial interval distributions” section).

### End-of-outbreak probability

The end-of-outbreak probability at time *t* of the outbreak (where the symptom onset time of the first recorded case is taken to be time 0, and times are given in whole numbers of days or weeks) is defined as the probability that no further cases occur after time *t*, conditional on the outbreak data up to and including time *t*.

Below, we describe five methods for estimating the end-of-outbreak probability, each of which is based on the branching process transmission model described above. Three of these methods (the simulation, enumerate, and MCMC methods) give the exact end-of-outbreak probability given disease incidence time series data (in addition to the offspring and serial interval distributions), and one method (the Nishiura method) gives an approximation to the end-of-outbreak probability. The remaining method (the traced method) gives the exact end-of-outbreak probability conditional on the disease incidence time series and the outbreak transmission tree.

In the below equations, disease incidence time series data are characterized by the total number, *n*, of cases up to the current time, *t*, and their symptom onset times, **τ** = (τ_1_, τ_2_, …, τ*_n_*). The outbreak transmission tree is characterized by the vector ***r*** = (*r*_1_, …, *r_n_*), where *r_i_* denotes the identity of the infector of individual *i* (and we define *r*_1_ = 0 to denote that the index case was an imported case).

#### Traced method

If the outbreak transmission tree is known up to and including time *t*, the exact end-of-outbreak probability under the assumed transmission model can be calculated (*5*) and is given by

Prob(outbreak over∣n,τ,r)=∏i=1np(ai)∑l=0∞(ai+ll)1−F(t−τi)lp(ai+l)=∏i=1n1−q1−F(t−τi)(k+ai) (1)

where the second equality applies for a negative binomial offspring distribution (as parameterized above), *a_i_* is the number of secondary transmissions generated to date by individual *i*, and all other notation is defined above. This expression is derived in the Supplementary Materials.

#### Enumerate method

If only disease incidence data are available, then an expression for the end-of-outbreak probability can be obtained by conditioning on the unknown transmission tree

$$\begin{array}{c} \text{Prob}\left(\text{outbreak over}|n,\tau \right)=\\ \sum_{r}\text{Prob}\left(\text{outbreak over}|n,\tau ,r\right)\times \text{Prob}\left(r|n,\tau \right) \end{array}$$ (2)

Here, the sum is taken over all transmission trees that are consistent with the disease incidence data; Prob(outbreak over∣*n*, **τ**, ***r***) is the end-of-outbreak probability for the traced method (Eq. 1 above); and Prob(***r***∣*n*, **τ**) is the likelihood of the transmission tree specified by ***r*** given the disease incidence time series data (an expression for the likelihood is derived in the Supplementary Materials—eqs. S14 and S15).

The enumerate method involves directly evaluating the sum in Eq. 2 by enumerating all possible transmission trees that are consistent with the disease incidence data.

#### MCMC method

Instead of calculating the sum in Eq. 2 directly, the MCMC method involves calculating the end-of-outbreak probability by using data augmentation MCMC to obtain a sample of possible transmission trees from the likelihood, Prob(***r***∣*n*, **τ**). Specifically, a version of the Metropolis-Hastings algorithm is used, where in each MCMC iteration, an augmented transmission tree is updated by proposing a new candidate infector for one randomly chosen individual. Equation 1 is then used to calculate the end-of-outbreak probability given the augmented transmission data for each MCMC iteration. An overall end-of-outbreak probability estimate (which is equivalent to Eq. 2 when a large number of MCMC iterations are used) is then obtained by calculating the average of the estimates from the individual iterations (after burn-in and thinning). The distribution of end-of-outbreak probability estimates from different MCMC iterations can also be used to quantify uncertainty in the end-of-outbreak probability depending on the exact transmission tree, leading to the credible intervals shown in Figs. 2 and 3.

Details of the MCMC algorithm are given in the Supplementary Materials. When we used the MCMC method to calculate the end-of-outbreak probability (at an individual time point), we carried out 10,000,000 total MCMC iterations, of which we discarded the first 2,000,000 iterations (burn-in) and then retained only one in every 1000 subsequent iterations (thinning). In all our analyses of either simulated or real-world data (except for the analysis of the largest simulated dataset—fig. S2C), this gave an estimated effective sample size correcting for autocorrelation (*33*) [calculated using the ArviZ Python package (*34*)] of at least 1000 from an overall sample of 8000 end-of-outbreak probability values in individual MCMC iterations (after burn-in and thinning). For the largest simulated dataset only, we carried out additional MCMC iterations to ensure that an estimated effective sample size of at least 1000 was achieved at each time point, as detailed in the caption to fig. S2.

#### Simulation method

The simulation method involves repeatedly simulating the forward branching process transmission model until a specified number of simulations have been obtained in which the simulated data match the recorded data up to the current time, *t* (we obtained 10,000 matching simulations whenever we used this method). The end-of-outbreak probability can then be estimated as the proportion of matching simulations in which no cases occur following time *t*.

In scenarios in which the transmission tree is known, this method could, in principle, be extended to match simulations to the recorded transmission tree. However, here we only considered matching simulations to disease incidence data (this is because our main aim when using the simulation method was to verify that, given identical data, the MCMC and simulation methods give very similar end-of-outbreak probability estimates).

#### Nishiura method

The Nishiura method is based on the following approximate formula for the end-of-outbreak probability given disease incidence data

$$\begin{array}{c} \text{Prob}\left(\text{outbreak over}|n,\tau \right)\approx \prod_{i=1}^{n}\sum_{y=0}^{\infty }p\left(y\right)F{\left(t-\tau_{i}\right)}^{y}=\\ \prod_{i=1}^{n}{\left[\frac{1-q}{1-qF\left(t-\tau_{i}\right)}\right]}^{k} \end{array}$$ (3)

where the second equality again applies for a negative binomial offspring distribution. This formula involves an assumption that the probability that an existing infected individual generates future infections (after time *t*) is independent of the number of infections that the individual has already generated. However, this assumption does not always hold in the underlying transmission model—specifically, for a negative binomial offspring distribution, this assumption does not hold except in the limit *k* → ∞ in which transmission is not overdispersed (this limit corresponds to a Poisson offspring distribution). Therefore, the Nishiura method only provides an approximation to the end-of-outbreak probability (even if the assumed transmission model is correct) (*5*).

### EVD offspring and serial interval distributions

In our analyses considering both simulated and real-world data, we assumed the following probability distributions characterizing EVD transmission:

1) A negative binomial offspring distribution with mean *R* = 0.95 and dispersion parameter *k* = 0.18 (*27*).

2) A gamma-distributed continuous serial interval (i.e., distribution of intervals between the precise symptom onset times of infector-infectee pairs) with mean 15.3 days and SD 9.3 days (*29*, *35*). We discretized this distribution using the method described in (*36*) to obtain a daily discrete serial interval distribution, ensuring a strictly positive serial interval by reassigning probability mass from 0 days to 1 day. In addition, when we considered weekly simulated data (Fig. 2), we used the same continuous distribution in units of weeks (i.e., a gamma distribution with mean 2.2 weeks and SD 1.3 weeks) and used the same discretization method to obtain a weekly discrete serial interval distribution.

The distributions described above are shown in the Supplementary Materials (fig. S1), and the offspring and daily serial interval distributions are also shown in Fig. 1 [inputs (ii) and (iii) in (A), respectively].

### Simulation study

We generated synthetic EVD outbreak data by simulating the assumed branching process transmission model using the EVD offspring and serial interval distributions described above. Specifically, we used the weekly serial interval distribution to generate datasets with weekly disease incidence data (Fig. 2), and the daily serial interval distribution to generate datasets with daily data (fig. S2). In addition, we considered datasets (with daily data) in which a single additional imported case was assumed to occur after a specified interval (14, 21, or 28 days) following the (otherwise) final case in a simulated outbreak (with no further cases occurring; fig. S4).

### EVD outbreak case studies

We considered real-world data from two past EVD outbreaks in the DRC. The first outbreak occurred in the Likati health zone in 2017 and comprised eight EVD cases occurring between 27 March and 11 May (*16*, *17*). The outbreak was declared over on 2 July, 42 days after the final case recovered (*16*). Daily disease incidence data (Fig. 3A) and the outbreak transmission tree (fig. S5) were available for this outbreak (*17*). For the purposes of our analyses, we shifted the symptom onset date of one case (ID 4 in fig. S5) to later than the date reported in (*17*) (24 April to 1 May) to avoid a 0-day serial interval. We did this because a 0-day serial interval is highly unlikely for EVD as transmission is typically only possible after symptom onset (*31*). Furthermore, we note that this later symptom onset date is consistent with epidemiological investigations undertaken at the time that suggest that individual ID 4 developed symptoms in May.

The second outbreak occurred in Équateur province in 2020 and comprised 130 EVD cases occurring between 9 May and 12 September (*18*–*20*). The outbreak was declared over on 18 November, 42 days after the final case recovered (*18*). Daily disease incidence data were available (*20*) (Fig. 3B).
