# Supplementary material for: Optimizing the timing of an end-of-outbreak declaration: Ebola virus disease in the Democratic Republic of the Congo
Source: Sci Adv. 2024 Jul 3;10(27):eado7576. doi: 10.1126/sciadv.ado7576 (PMC11221504; doi:10.1126/sciadv.ado7576)
Supplement: Supplementary file 1 — Supplementary Text Figs. S1 to S7 [file sciadv.ado7576_sm.pdf]

Science Advances
AAAS

Supplementary Materials for

Optimizing the timing of an end-of-outbreak declaration: Ebola virus disease in the Democratic Republic of the Congo

William S. Hart *et al.*

Corresponding author: William S. Hart, william.hart@maths.ox.ac.uk

Sci. Adv. **10**, eado7576 (2024)
DOI: 10.1126/sciadv.ado7576

This PDF file includes:

Supplementary Text
Figs. S1 to S7

Supplementary Text

Notation

We use the following notation throughout the Supplementary Text:

- $p(y)$ is the probability mass function of the offspring distribution (for $y = 0,1,2,\ldots$). When considering a negative binomial offspring distribution with mean R and dispersion parameter k, we take $p(y) = \frac{\Gamma(k+y)}{y!\Gamma(k)} q^y (1-q)^k$, where $q = R/(R+k)$.
- $f(x)$ is the probability mass function of the discrete (daily or weekly) serial interval distribution (for $x = 1,2,\ldots$ days or weeks), and $F(x)$ is the corresponding cumulative distribution function.
- t is the time up to (and including) which data are available (at which the end-of-outbreak probability is to be estimated), where the symptom onset time of the first case is taken to be time 0, and all times are either in whole days or weeks (dependent on whether daily or weekly disease incidence data are available).
- n is the number of recorded cases to date.
- The recorded cases (up to and including time t) are labelled with integer IDs $i = 1,2,\ldots,n$, ordered by symptom onset time.
- τ_i is the symptom onset time of case i, and we write $\boldsymbol{\tau} = (\tau_1, \tau_2, \ldots, \tau_n)$.
- r_i is the ID of the (recorded or estimated) infector of case i, and we write $\boldsymbol{r} = (r_1, \ldots, r_n)$. We define $r_1 = 0$ to specify that the index case was an imported case, and we assume for simplicity that all other cases arose as a result of local transmission (note that when considering multiple imported cases in Fig. S4, we assumed that some of our results remain valid in this scenario, as described in the caption to that figure).
- $z_{i,s}$ is the number of cases infected by case i who go on to develop symptoms at time $0 \leq s \leq t$ (under a recorded or estimated transmission tree). We write Z to denote the $(n \times (t+1))$ matrix with entries $z_{i,s}$, and $\boldsymbol{z_i} = (z_{i,0}, \ldots, z_{i,t})$ for the sequence of case numbers generated by individual i, up to (and including) time t.
- a_i is the number of recorded transmissions generated by individual i up to (and including) time t, and we write $\boldsymbol{a} = (a_1, \ldots, a_n)$.
- Y_i is a random variable giving the total number of secondary cases ever generated by individual i, including those occurring after the current time, t, and we write $\boldsymbol{Y} = (Y_1, \ldots, Y_n)$.

Disease incidence data are characterised by the pair $(n, \boldsymbol{\tau})$, where the dependence on n is included to specify explicitly that exactly n cases have occurred up to time t. The transmission tree is characterised by the vector of infectors, $\boldsymbol{r}$.

Derivation of end-of-outbreak probability given the transmission tree

Here, we derive the expression for the end-of-outbreak probability given both disease incidence data and the outbreak transmission tree (recorded or estimated), up to and including the current time, t (i.e., the end-of-outbreak probability for the traced method – equation (1) in the main text).

We calculate:

$$\begin{aligned}
\text{Prob}(\text{outbreak over} \mid n, \boldsymbol{\tau}, \boldsymbol{r}) &= \text{Prob}(\boldsymbol{Y} = \boldsymbol{a} \mid n, \boldsymbol{\tau}, \boldsymbol{r}) \\
&= \text{Prob}(\boldsymbol{Y} = \boldsymbol{a} \mid \boldsymbol{\tau}, Z) \\
&= \prod_{i=1}^{n} \text{Prob}(Y_i = a_i \mid \boldsymbol{\tau}, Z) \\
&= \prod_{i=1}^{n} \text{Prob}(Y_i = a_i \mid \tau_i, \boldsymbol{z_i}) \\
&= \prod_{i=1}^{n} \frac{\text{Prob}(Y_i = a_i, \boldsymbol{z_i} \mid \tau_i)}{\text{Prob}(\boldsymbol{z_i} \mid \tau_i)}.
\end{aligned} \tag{S1}$$

Here, the second and fourth equalities follow because the probability of each recorded case generating future cases depends only on their symptom onset time and information on the transmissions they have generated to date, and the third equality follows by the assumption that transmissions occur according to a branching process.

Now, for integer $l \geq 0$, we have:

$$\begin{aligned}
\text{Prob}(Y_i = a_i + l, \boldsymbol{z_i} \mid \tau_i) &= \text{Prob}(\boldsymbol{z_i} \mid \tau_i, Y_i = a_i + l) \times \text{Prob}(Y_i = a_i + l \mid \tau_i) \\
&= \binom{a_i + l}{\boldsymbol{z_i}, l} \left(\prod_{s=0}^{t} f(s - \tau_i)^{z_{i,s}} \right) \left(1 - F(t - \tau_i)\right)^l p(a_i + l),
\end{aligned} \tag{S2}$$

where $f(s - \tau_i)^{z_{i,s}}$ gives the probability that $z_{i,s}$ specified secondary cases generated by i develop symptoms at time s (defining this term to equal 1 when both $f(s - \tau_i)$ and $z_{i,s}$ are zero), $(1 - F(t - \tau_i))^l$ gives the probability that l specified secondary cases develop symptoms after the current time, t, and the multinomial coefficient,

$$\binom{a_i + l}{\boldsymbol{z_i}, l} = \frac{(a_i + l)!}{z_{i,0}!\, z_{i,1}! \ldots z_{i,t}!\, l!} = \binom{a_i + l}{l} \times \binom{a_i}{\boldsymbol{z_i}}, \tag{S3}$$

gives the number of ways in which the $(a_i + l)$ total secondary cases can be divided into cases developing symptoms on each time up to time t and cases developing symptoms after time t. Therefore,

$$\text{Prob}(Y_i = a_i, \boldsymbol{z_i} \mid \tau_i) = \binom{a_i}{\boldsymbol{z_i}} \left(\prod_{s=0}^{t} f(s - \tau_i)^{z_{i,s}} \right) p(a_i), \tag{S4}$$

and

$$\begin{aligned}
\text{Prob}(\boldsymbol{z_i} \mid \tau_i) &= \sum_{l=0}^{\infty} \text{Prob}(Y_i = a_i + l, \boldsymbol{z_i} \mid \tau_i) \\
&= \binom{a_i}{\boldsymbol{z_i}} \left(\prod_{s=0}^{t} f(s - \tau_i)^{z_{i,s}} \right) \sum_{l=0}^{\infty} \binom{a_i + l}{l} \left(1 - F(t - \tau_i)\right)^l p(a_i + l).
\end{aligned} \tag{S5}$$

Substituting equations (S4) and (S5) into equation (S1) then gives

$$\text{Prob}(\text{outbreak over} \mid n, \boldsymbol{\tau}, \boldsymbol{r}) = \prod_{i=1}^{n} \frac{p(a_i)}{\sum_{l=0}^{\infty} \binom{a_i+l}{l} \left(1 - F(t - \tau_i)\right)^l p(a_i + l)}. \tag{S6}$$

Finally, for a negative binomial offspring distribution,

$$\begin{aligned}
&\sum_{l=0}^{\infty}\binom{a_i+l}{l}\big(1-F(t-\tau_i)\big)^l p(a_i+l) \\
&=\sum_{l=0}^{\infty}\frac{(a_i+l)!}{l!\,a_i!}\big(1-F(t-\tau_i)\big)^l\frac{\Gamma(k+a_i+l)}{(a_i+l)!\,\Gamma(k)}q^{a_i+l}(1-q)^k \\
&=\frac{\Gamma(k+a_i)}{a_i!\,\Gamma(k)}q^{a_i}(1-q)^k\Big(1-q\big(1-F(t-\tau_i)\big)\Big)^{-(k+a_i)} \\
&\quad\times\sum_{l=0}^{\infty}\frac{\Gamma\big((k+a_i)+l\big)}{l!\,\Gamma(k+a_i)}\Big(q\big(1-F(t-\tau_i)\big)\Big)^l\Big(1-q\big(1-F(t-\tau_i)\big)\Big)^{(k+a_i)} \\
&=p(a_i)\Big(1-q\big(1-F(t-\tau_i)\big)\Big)^{-(k+a_i)},
\end{aligned} \tag{S7}$$

where the final equality is obtained because the expression inside the sum on the previous line is the probability mass function of a negative binomial distribution. Substituting equation (S7) into equation (S6), we obtain

$$\text{Prob}(\text{outbreak over} \mid n,\boldsymbol{\tau},\boldsymbol{r}) = \prod_{i=1}^{n}\big(1-q(1-F(t-\tau_i))\big)^{(k+a_i)}. \tag{S8}$$

Derivation of likelihood of transmission tree given disease incidence data

Here, we derive the likelihood, $\text{Prob}(\boldsymbol{r} \mid n,\boldsymbol{\tau})$, of the transmission tree characterised by $\boldsymbol{r}$ given disease incidence data characterised by $(n,\boldsymbol{\tau})$, which is used in the MCMC and enumerate methods for calculating the end-of-outbreak probability.

We first consider the probability of transmission frequency matrix Z given the disease incidence data (where multiple distinct transmission trees may give rise to the same Z, as quantified later):

$$\begin{aligned}
\text{Prob}(Z \mid n,\boldsymbol{\tau}) &= \frac{\text{Prob}(Z,n \mid \boldsymbol{\tau})}{\text{Prob}(n \mid \boldsymbol{\tau})} \\
&\propto \text{Prob}(Z,n \mid \boldsymbol{\tau}) \\
&= \text{Prob}(Z \mid \boldsymbol{\tau}) \\
&= \text{Prob}(\boldsymbol{z_n} \mid \boldsymbol{z_1},\dots,\boldsymbol{z_{n-1}},\boldsymbol{\tau})\text{Prob}(\boldsymbol{z_1},\dots,\boldsymbol{z_{n-1}} \mid \boldsymbol{\tau}) \\
&= \text{Prob}(\boldsymbol{z_n} \mid \tau_n)\text{Prob}(\boldsymbol{z_1},\dots,\boldsymbol{z_{n-1}} \mid \boldsymbol{\tau}) \\
&= \text{Prob}(\boldsymbol{z_n} \mid \tau_n)\times\frac{\text{Prob}(\boldsymbol{z_1},\dots,\boldsymbol{z_{n-1}},\tau_n \mid \tau_1,\dots,\tau_{n-1})}{\text{Prob}(\tau_n \mid \tau_1,\dots,\tau_{n-1})} \\
&\propto \text{Prob}(\boldsymbol{z_n} \mid \tau_n)\text{Prob}(\boldsymbol{z_1},\dots,\boldsymbol{z_{n-1}},\tau_n \mid \tau_1,\dots,\tau_{n-1}) \\
&= \text{Prob}(\boldsymbol{z_n} \mid \tau_n)\text{Prob}(\boldsymbol{z_1},\dots,\boldsymbol{z_{n-1}} \mid \tau_1,\dots,\tau_{n-1}) \\
&\propto \cdots \propto \prod_{i=1}^{n}\text{Prob}(\boldsymbol{z_i} \mid \tau_i),
\end{aligned} \tag{S9}$$

where each constant of proportionality depends only on n and $\boldsymbol{\tau}$. Using equation (S5), we then have

$$\text{Prob}(Z \mid n, \boldsymbol{\tau}) \propto \prod_{i=1}^{n} \binom{a_i}{\boldsymbol{z}_i} \left(\prod_{s=0}^{t} f(s-\tau_i)^{z_{i,s}} \right) \sum_{l=0}^{\infty} \binom{a_i + l}{l} \big(1 - F(t-\tau_i)\big)^l p(a_i + l). \tag{S10}$$

Now, the likelihood is given by

$$\begin{aligned} \text{Prob}(\boldsymbol{r} \mid n, \boldsymbol{\tau}) &= \text{Prob}(\boldsymbol{r}, Z \mid n, \boldsymbol{\tau}) \\ &= \text{Prob}(\boldsymbol{r} \mid n, \boldsymbol{\tau}, Z)\text{Prob}(Z \mid n, \boldsymbol{\tau}). \end{aligned} \tag{S11}$$

We note that the number of transmission trees that would give rise to specified n, $\boldsymbol{\tau}$ and Z is

$$\prod_{s=0}^{t} \binom{\sum_{i=1}^{n} z_{i,s}}{z_{1,s}, \dots, z_{n,s}}, \tag{S12}$$

and each of these transmission trees is equally likely. Therefore, we have

$$\begin{aligned} \text{Prob}(\boldsymbol{r} \mid n, \boldsymbol{\tau}, Z) &= \frac{1}{\prod_{s=0}^{t} \binom{\sum_{i=1}^{n} z_{i,s}}{z_{1,s}, \dots, z_{n,s}}} \\ &\propto \prod_{i=1}^{n} \prod_{s=0}^{t} z_{i,s}!, \end{aligned} \tag{S13}$$

where the constant of proportionality again depends only on n and $\boldsymbol{\tau}$ (since $\sum_{i=1}^{n} z_{i,s}$ is simply the total number of cases arising at time s). Substituting equations (S10) and (S13) into equation (S11) then gives

$$\text{Prob}(\boldsymbol{r} \mid n, \boldsymbol{\tau}) \propto \prod_{i=1}^{n} a_i! \left(\prod_{s=0}^{t} f(s-\tau_i)^{z_{i,s}} \right) \sum_{l=0}^{\infty} \binom{a_i + l}{l} \big(1 - F(t-\tau_i)\big)^l p(a_i + l). \tag{S14}$$

Finally, for a negative binomial offspring distribution, substituting equation (S7) into equation (S14) gives

$$\text{Prob}(\boldsymbol{r} \mid n, \boldsymbol{\tau}) \propto \prod_{i=1}^{n} a_i! \left(\prod_{s=0}^{t} f(s-\tau_i)^{z_{i,s}} \right) p(a_i) \Big(1 - q\big(1 - F(t-\tau_i)\big)\Big)^{-(k+a_i)}. \tag{S15}$$

In the MCMC method for calculating the end-of-outbreak probability, the exact constant of proportionality in equation (S15) is not required to sample possible transmission trees from the likelihood, $\text{Prob}(\boldsymbol{r} \mid n, \boldsymbol{\tau})$. On the other hand, when we used the enumerate method to calculate the end-of-outbreak probability, we used equation (S15) to calculate the relative likelihoods of all possible transmission trees, and then normalised these likelihood values to ensure that they sum to one.

Details of MCMC algorithm

The algorithm used in the MCMC method for calculating the end-of-outbreak probability given disease incidence data (characterised by the pair $(n, \boldsymbol{\tau})$) is detailed below. We denote the total number of MCMC iterations by $M = M_B + M_T M_K$, where M_K is the number of iterations retained after discarding the first M_B iterations (to allow the chain to converge to its stationary distribution – burn-in) and then keeping only one in M_T subsequent iterations (to reduce autocorrelation – thinning).

The MCMC algorithm, which is a version of the Metropolis-Hastings algorithm, involves the following steps:

1. Initialise the augmented transmission tree, $\boldsymbol{r} = \boldsymbol{r}^{(0)}$ (we sampled the identity of the infector of each locally infected case according to the serial interval distribution, as described below).
2. For each MCMC iteration, $m = 1,2,\ldots,M$, do the following:
    a. Sample a new transmission tree, $\boldsymbol{r}^{(\mathrm{prop})}$, from the proposal distribution, denoted $Q\left(\boldsymbol{r}^{(\mathrm{prop})} \mid n, \boldsymbol{\tau}, \boldsymbol{r}^{(m-1)}\right)$. Here, we describe the MCMC algorithm for a general proposal distribution; the specific proposal distribution used in our analyses is given below.
    b. Calculate the acceptance probability,
    $$\alpha = \min\left(\frac{\mathrm{Prob}\left(\boldsymbol{r}^{(\mathrm{prop})} \mid n, \boldsymbol{\tau}\right) Q\left(\boldsymbol{r}^{(m-1)} \mid n, \boldsymbol{\tau}, \boldsymbol{r}^{(\mathrm{prop})}\right)}{\mathrm{Prob}\left(\boldsymbol{r}^{(m-1)} \mid n, \boldsymbol{\tau}\right) Q\left(\boldsymbol{r}^{(\mathrm{prop})} \mid n, \boldsymbol{\tau}, \boldsymbol{r}^{(m-1)}\right)}, 1\right), \tag{S16}$$
    where the likelihood, $\mathrm{Prob}(\boldsymbol{r} \mid n, \boldsymbol{\tau})$, is given in equation (S15) above (a simplified version of equation (S16) under our specific choice of proposal distribution is given below).
    c. Generate a random number, u, uniformly distributed between 0 and 1.
    d. If $u \leq \alpha$, set $\boldsymbol{r}^{(m)} = \boldsymbol{r}^{(\mathrm{prop})}$ (i.e., accept the proposed transmission tree). Otherwise, set $\boldsymbol{r}^{(m)} = \boldsymbol{r}^{(m-1)}$.
    e. If iteration m is kept after burn-in and thinning, use the traced method (equation (1) in the main text) to calculate the end-of-outbreak probability, $\mathrm{Prob}\left(\text{outbreak over} \mid n, \boldsymbol{\tau}, \boldsymbol{r}^{(m)}\right)$, given the current transmission tree.
3. Calculate the overall end-of outbreak probability estimate,

$$\mathrm{Prob}(\text{outbreak over} \mid n, \boldsymbol{\tau}) \approx \frac{1}{M_K} \sum_{m_K=1}^{M_K} \mathrm{Prob}\left(\text{outbreak over} \mid n, \boldsymbol{\tau}, \boldsymbol{r}^{(M_B + m_K M_T)}\right). \tag{S17}$$

<u>Choice of proposal distribution and algebraic simplification</u>

In each MCMC iteration, m, we generated a proposed transmission tree, $\boldsymbol{r}^{(\mathrm{prop})}$, by resampling the imputed infector of a single locally infected case, i (with $\boldsymbol{r}^{(\mathrm{prop})}$ otherwise identical to $\boldsymbol{r}^{(m-1)}$). Specifically, individual i was selected uniformly at random from the set of all locally infected cases. The proposed infector, $r_i^{(\mathrm{prop})} = l$, was then sampled according to the serial interval distribution, i.e., individual l was chosen with probability

$$\frac{f(\tau_i - \tau_l)}{\sum_{\tilde{l}=1}^{i-1} f(\tau_i - \tau_{\tilde{l}})}, \tag{S18}$$

independently of the imputed infector, j, from the previous MCMC step. We found this choice to be more efficient than sampling a proposed infector uniformly at random (out of the set of all possible infectors). Note that we assumed that $f(0) = 0$, so that an infector who developed symptoms at the same time point as individual i is never selected. The initial transmission tree, $\boldsymbol{r}^{(0)}$, was also generated by sampling an infector for each locally infected case in this manner.

Now, defining i, j and l as in the previous paragraph, we have

$$\frac{Q\left(\boldsymbol{r}^{(m-1)} \mid n, \boldsymbol{\tau}, \boldsymbol{r}^{(\mathrm{prop})}\right)}{Q(\boldsymbol{r}^{(\mathrm{prop})} \mid n, \boldsymbol{\tau}, \boldsymbol{r}^{(m-1)})} = \frac{f\left(\tau_i - \tau_j\right)}{f(\tau_i - \tau_l)}. \tag{S19}$$

Further, we note that under a negative binomial offspring distribution, only the jth and l^{th} terms in the product defining the likelihood in equation (S15) differ between the previous and proposed transmission trees. Specifically, for $l \neq j$,

$$\frac{\mathrm{Prob}\left(\boldsymbol{r}^{(\mathrm{prop})} \mid n, \boldsymbol{\tau}\right)}{\mathrm{Prob}\left(\boldsymbol{r}^{(m-1)} \mid n, \boldsymbol{\tau}\right)} = \frac{f(\tau_i - \tau_l)}{f\left(\tau_i - \tau_j\right)} \times \frac{L_{j-}^{(m-1)} L_{l+}^{(m-1)}}{L_j^{(m-1)} L_l^{(m-1)}}, \tag{S20}$$

Here, $L_j^{(m-1)}$ denotes the components of the jth term in the likelihood of the previous transmission tree (as given by equation (S15)) that do not involve realised serial intervals, i.e.,

$$L_j^{(m-1)} = a_j^{(m-1)}!\, p\left(a_j^{(m-1)}\right)\left(1 - q\left(1 - F\left(t - \tau_j\right)\right)\right)^{-\left(k + a_j^{(m-1)}\right)}, \tag{S21}$$

where $a_j^{(m-1)}$ is the total number of secondary cases generated to date by individual j under the previous transmission tree. $L_{j\pm}^{(m-1)}$ is the corresponding quantity to $L_j^{(m-1)}$ but with $a_j^{(m-1)}$ replaced by $a_j^{(m-1)} \pm 1$.

Finally, combining equations (S19) and (S20) and simplifying, the acceptance ratio in equation (S16) is given (for $l \neq j$) by

$$\frac{\mathrm{Prob}\left(\boldsymbol{r}^{(\mathrm{prop})} \mid n, \boldsymbol{\tau}\right) Q\left(\boldsymbol{r}^{(m-1)} \mid n, \boldsymbol{\tau}, \boldsymbol{r}^{(\mathrm{prop})}\right)}{\mathrm{Prob}\left(\boldsymbol{r}^{(m-1)} \mid n, \boldsymbol{\tau}\right) Q(\boldsymbol{r}^{(\mathrm{prop})} \mid n, \boldsymbol{\tau}, \boldsymbol{r}^{(m-1)})} = \frac{\left(k + a_l^{(m-1)}\right)\left(1 - q\left(1 - F\left(t - \tau_j\right)\right)\right)}{(k + a_j^{(m-1)} - 1)\left(1 - q\left(1 - F(t - \tau_l)\right)\right)}. \tag{S22}$$

Supplementary Figures

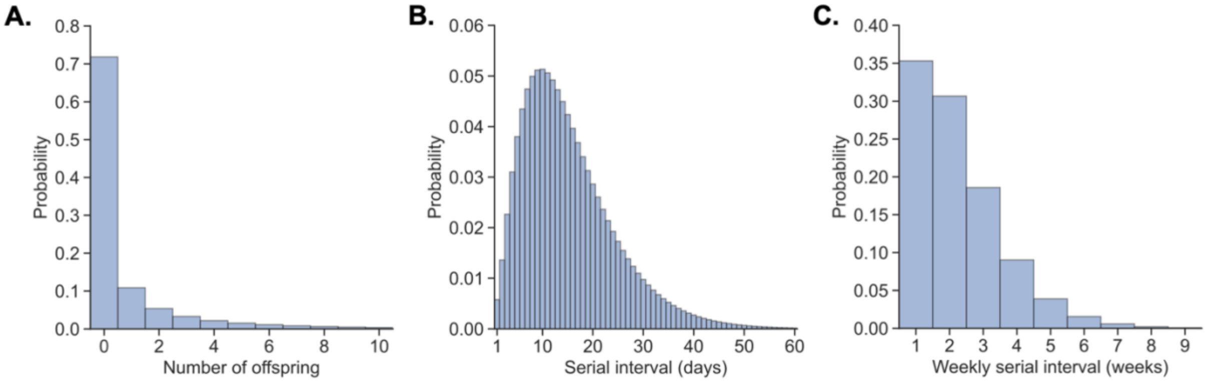

Fig. S1. Assumed offspring and serial interval distributions characterising EVD transmission. A. Offspring distribution. B. Daily serial interval distribution. C. Weekly serial interval distribution used in the simulation study when considering weekly disease incidence data. The choice of these distributions is described in Materials and Methods in the main text.

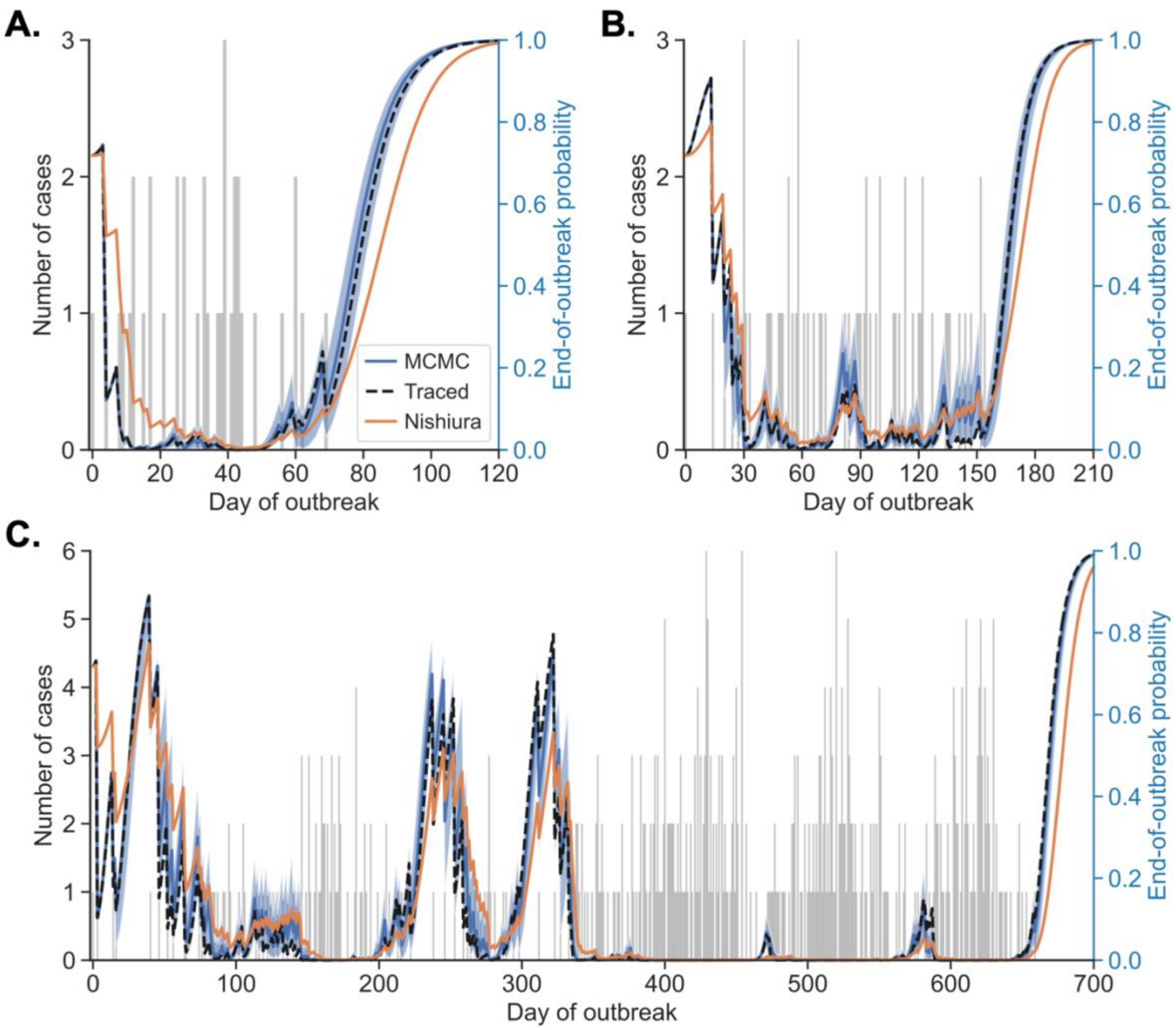

Fig. S2. End-of-outbreak probability estimates for simulated datasets with daily data. A. Disease incidence data (grey bars; values are shown on the left y-axis) and end-of-outbreak probability estimates obtained using the MCMC, traced and Nishiura methods (values are shown on the right y-axis) for the first simulated dataset with daily disease incidence data (note that this is an entirely different dataset from that considered in Fig. 2A of the main text). The blue shaded region indicates 95% credible intervals of end-of-outbreak probability estimates obtained in individual MCMC iterations. B. Equivalent panel for the second simulated dataset with daily data. C. Equivalent panel for the third simulated dataset with daily data. In panel C only, for each end-of-outbreak probability calculation using the MCMC method, 20,000,000 total MCMC iterations were carried out, of which the first 4,000,000 iterations were discarded (burn-in) and then only one in every 2,000 subsequent iterations were retained (thinning); the number of MCMC iterations used in panels A-B is given in Materials and Methods in the main text.

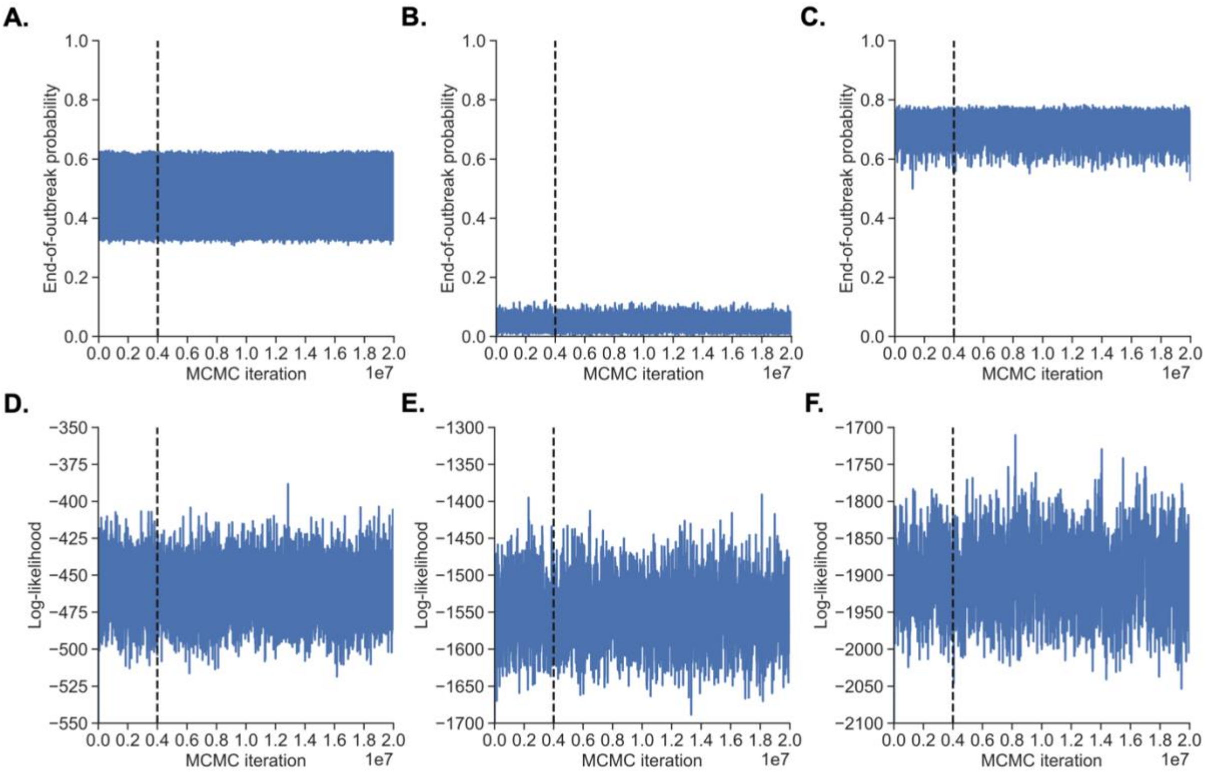

Fig. S3. Example MCMC trace plots from the simulation study. A-C. Trace plots for calculations of the end-of-outbreak probability for the simulated outbreak shown in Fig. S2C using the MCMC method with data from up to day 325 (A), day 575 (B) and day 675 (C) of the outbreak. The end-of-outbreak probability conditional on the current augmented transmission tree is plotted for each of the one in 2,000 MCMC iterations retained after thinning (note that the x-axis numbering includes iterations removed after thinning). The initial burn-in period of 4,000,000 iterations is indicated by the vertical black dashed line. Estimated effective sample sizes for the chains shown in panels A-C (each from a sample size of 8,000 iterations retained after burn-in and thinning) are 6,700, 3,300 and 3,100, respectively (to two significant figures). D-F. Corresponding trace plots of the log-likelihood.

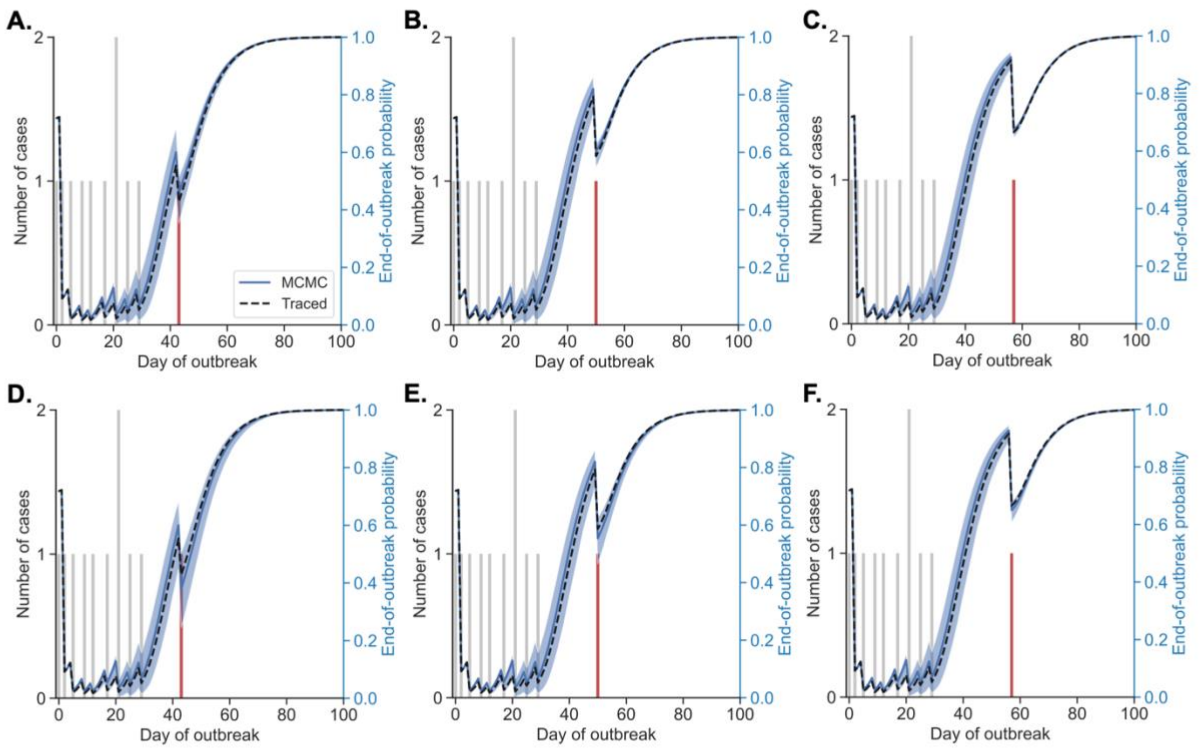

Fig. S4. End-of-outbreak probability estimates for simulated datasets with an additional imported case. A. Disease incidence data (bars; values are shown on the left y-axis) are shown for a dataset in which a final, imported, case (shown in red) occurs 14 days after the penultimate case. Estimated end-of-outbreak probabilities (lines; values are shown on the right y-axis) are shown for the MCMC method, assuming the final case is known to be an imported case, and for the traced method. Note that we assumed that the likelihood expression in equation (S15) remains valid with an additional imported case (the end-of-outbreak probability formula for the traced method in equation (1) was shown to hold in this scenario in (5)). B-C. Equivalent panels to A except with the final imported case occurring 21 days (B) or 28 days (C) following the penultimate case (the datasets shown in each panel are identical other than the timing of the final case). D-F. Equivalent panels to A-C, except assuming the final case to have arisen as a result of local transmission from one of the previous cases when using the MCMC method to estimate the end-of-outbreak probability.

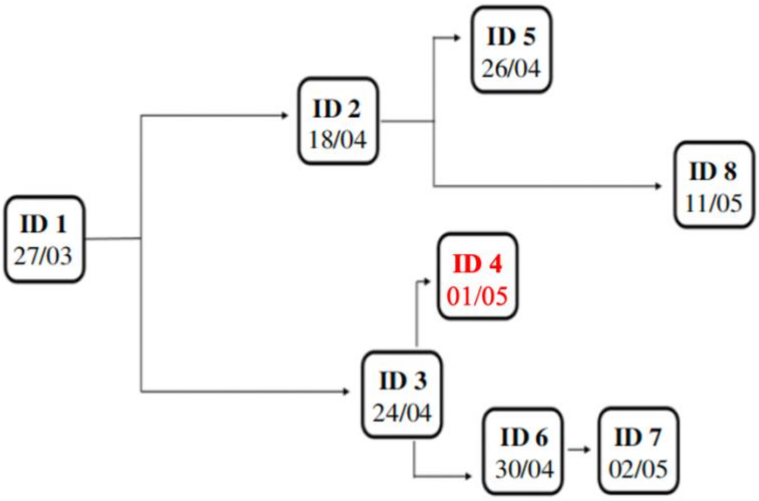

Fig. S5. Transmission tree for 2017 Likati EVD outbreak. Dates are in DD/MM format (all 2017). Note that in our analyses, we shifted the symptom date of one case (ID 4) to a later date than the date reported in (17) to avoid a zero-day serial interval (24 April to 1 May). This later symptom onset date is consistent with epidemiological investigations undertaken at the time that suggest that individual ID 4 developed symptoms in May.

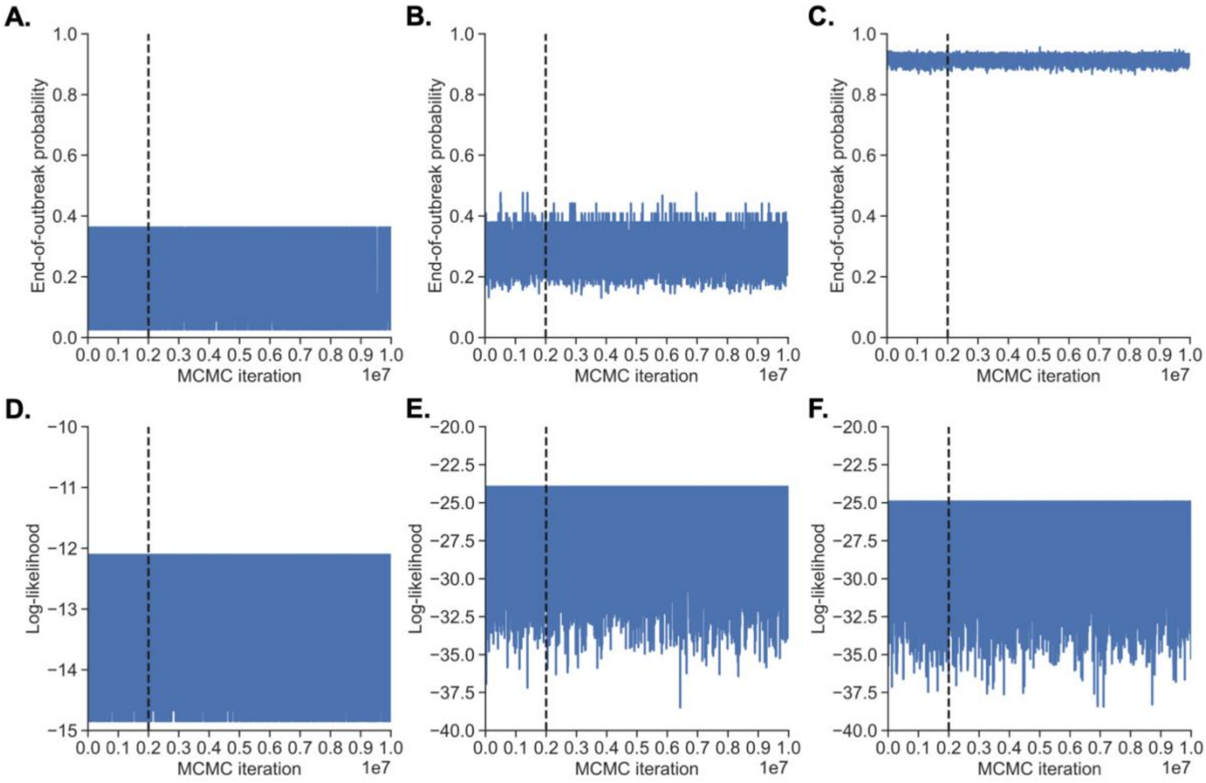

Fig. S6. Example MCMC trace plots for the 2017 Likati health zone EVD outbreak. A-C. Trace plots for calculations of the end-of-outbreak probability using the MCMC method with data from up to day 30 (A), day 50 (B) and day 70 (C) of the outbreak. The end-of-outbreak probability conditional on the current augmented transmission tree is plotted for each of the one in 1,000 MCMC iterations retained after thinning (note that the x-axis numbering includes iterations removed after thinning). The initial burn-in period of 2,000,000 iterations is indicated by the vertical black dashed line. Estimated effective sample sizes for the chains shown in panels A-C (each from a sample size of 8,000 iterations retained after burn-in and thinning) are 7,900, 8,300 and 7,900, respectively (to two significant figures). D-F. Corresponding trace plots of the log-likelihood.

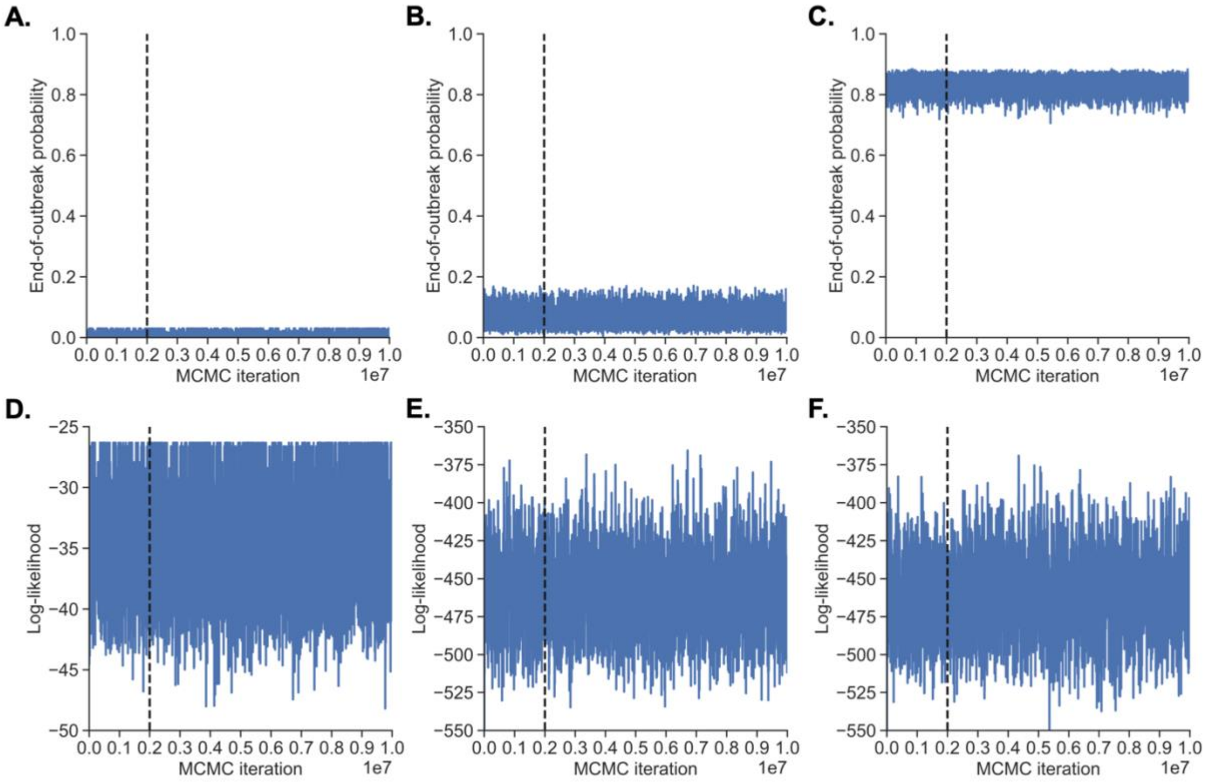

Fig. S7. Example MCMC trace plots for the 2020 Équateur province EVD outbreak. A-C. Trace plots for calculations of the end-of-outbreak probability using the MCMC method with data from up to day 25 (A), day 130 (B) and day 150 (C) of the outbreak. The end-of-outbreak probability conditional on the current augmented transmission tree is plotted for each of the one in 1,000 MCMC iterations retained after thinning (note that the x-axis numbering includes iterations removed after thinning). The initial burn-in period of 2,000,000 iterations is indicated by the vertical black dashed line. Estimated effective sample sizes for the chains shown in panels A-C (each from a sample size of 8,000 iterations retained after burn-in and thinning) are 7,600, 4,300 and 3,900, respectively (to two significant figures). D-F. Corresponding trace plots of the log-likelihood.
